# Mass spectrometry imaging of natural carbonyl products directly from agar-based microbial interactions using 4-APEBA derivatization

**DOI:** 10.1128/msystems.00803-23

**Published:** 2023-12-08

**Authors:** Dušan Veličković, Kevin J. Zemaitis, Arunima Bhattacharjee, Christopher R. Anderton

**Affiliations:** 1 Environmental Molecular Sciences Laboratory, Pacific Northwest National Laboratory, Richland, Washington, USA; Max Planck Institute for Marine Microbiology, Bremen, Germany

**Keywords:** MALDI, metabolomics, carboxylic acids, aldehydes, ketones, *Bacillus subtilis*, *Fusarium*

## Abstract

Aliphatic carboxylic acids, aldehydes, and ketones play diverse roles in microbial adaptation to their microenvironment, from excretion as toxins to adaptive metabolites for membrane fluidity. However, the spatial distribution of these molecules throughout biofilms and how microbes in these environments exchange these molecules remain elusive for many of these bioactive species due to inefficient molecular imaging strategies. Herein, we apply on-tissue chemical derivatization (OTCD) using 4-(2-((4-bromophenethyl)dimethylammonio)ethoxy)benzenaminium dibromide (4-APEBA) on a co-culture of a soil bacterium (*Bacillus subtilis* NCIB 3610) and fungus (*Fusarium* sp. DS 682) grown on agar as our model system. Using matrix-assisted laser desorption/ionization mass spectrometry imaging (MALDI-MSI), we spatially resolved more than 300 different metabolites containing carbonyl groups within this model system. Various spatial patterns are observable in these species, which indicate possible extracellular or intercellular processes of the metabolites and their up- or downregulation during microbial interaction. The unique chemistry of our approach allowed us to bring additional confidence in accurate carbonyl identification, especially when multiple isomeric candidates were possible, and this provided the ability to generate hypotheses about the potential role of some aliphatic carbonyls in this *B. subtilis*/*Fusarium* sp. interaction. The results shown here demonstrate the utility of 4-ABEBA-based OTCD MALDI-MSI in probing interkingdom interactions directly from microbial co-cultures, and these methods will enable future microbial interaction studies with expanded metabolic coverage.

**IMPORTANCE:**

The metabolic profiles within microbial biofilms and interkingdom interactions are extremely complex and serve a variety of functions, which include promoting colonization, growth, and survival within competitive and symbiotic environments. However, measuring and differentiating many of these molecules, especially in an *in situ* fashion, remains a significant analytical challenge. We demonstrate a chemical derivatization strategy that enabled highly sensitive, multiplexed mass spectrometry imaging of over 300 metabolites from a model microbial co-culture. Notably, this approach afforded us to visualize over two dozen classes of ketone-, aldehyde-, and carboxyl-containing molecules, which were previously undetectable from colonies grown on agar. We also demonstrate that this chemical derivatization strategy can enable the discrimination of isobaric and isomeric metabolites without the need for orthogonal separation (e.g., online chromatography or ion mobility). We anticipate that this approach will further enhance our knowledge of metabolic regulation within microbiomes and microbial systems used in bioengineering applications.

## INTRODUCTION

Mass spectrometry imaging (MSI) is becoming an established technique for exploring the nature and diversity of the chemical compounds produced in microbial systems (1–3). It has been extensively applied in understanding biochemical processes in microbial and host-microbe interactions (2), biofilm formation (4), and microbial phenotyping (5). Within the literature, spatial probing via MSI for retrieving localized metabolic signatures is commonly performed on microbial systems grown on agar or other solid growing media, including other organisms (6). While there are numerous types of ionization modalities and mass spectrometry approaches, such as nanospray desorption electrospray ionization (nanoDESI) (7), laser ablation electrospray ionization (LAESI) (8), liquid extraction surface analysis (LESA) (9), and ultrahigh lateral resolution secondary ion mass spectrometry (SIMS) (10), which have been used to chemically image microbial samples, matrix-assisted laser desorption/ionization (MALDI) methods are most broadly implemented, in part, due to their robustness, reproducibility, high spatial resolution, and wide molecular coverage (1, 11–14).

While there are many classes of metabolites and small molecules that can be readily detected and annotated by MALDI-MSI (14), the ability to obtain comprehensive detection of natural acidic compounds, including aliphatic carboxylates and carbonyls, which play diverse roles in microbes (15–18) and are valuable additives in food, fragrances, and pharmaceuticals (15, 16), remains a significant challenge. For example, MALDI-MSI analyses of agar-based samples have been limited to the positive ionization modality where these classes of biomolecules ionize poorly. The sparse reporting of negative ionization mode analyses is presumed to be ascribed to the chemistry of the agar, which has a negative charge due to sulfate groups. This could dissipate charge during negative ion mode analysis and/or possibly limit the ability of MALDI matrices used in negative ionization mode to efficiently extract and co-crystalize with analytes on the agar surface. Perhaps this is, in part, a reason why others developed imprinting strategies to transfer colonies from agar to more suitable supports for negative ionization mode analysis (19). Nevertheless, even imprinting approaches have limitations, where transfer efficiency, molecular selectivity, and molecular relocation are notable issues for comprehensive molecular mapping (20).

Here, we provide a new MSI approach for mapping endogenous metabolites containing carbonyl groups from microbial systems. We applied our previously developed on-tissue chemical derivatization (OTCD) protocol to microbial cultures grown on agar as a proof of concept of this approach (21). There are a growing number of OTCD reagents and protocols that have been developed to increase the sensitivity and molecular coverage from mammalian and plant samples in MALDI-MSI (22–24). Our approach uses 4-(2-((4-bromophenethyl)dimethylammonio)ethoxy)benzenaminium dibromide (4-APEBA), which adds a permanent positive charge to carbonyl analytes, making them amenable to positive ionization mode analysis in MSI, which is especially useful for analyzing agar-based microbial colonies. Additionally, the bromine in 4-APEBA introduces a unique isotopic pattern to derivatized molecules, which can be exploited for more confident analyte annotation (21). We used this approach to study the interaction of the soil microbes *Bacillus subtilis* NCIB 3610 and *Fusarium* sp. DS 682 (25). Our results demonstrate that this approach enabled high-sensitivity analysis of over 300 microbially generated carbonyl-containing molecules directly from microbial cultures on agar plates. To our knowledge, this is the first demonstration of using an OTCD approach for MSI-based metabolic profiling of microbial systems.

## RESULTS AND DISCUSSION

Our results show that 4-APEBA-based OTCD of the *B. subtilis* NCIB 3610 and *Fusarium* sp. DS 682 interaction enabled confident chemical formula annotations of over 300 various carbonyls, whose putative structural annotations are proposed in Table S1 and are further classified in Fig. S1. In comparison, when we analyzed sample replicates without OTCD, in the negative ionization mode, using NEDC (*N*-(1-naphthyl)ethylenediamine dihydrochloride) as a MALDI matrix, less than 10 annotated molecular formulae were annotated with high confidence, and the list of annotations can be found on METASPACE (26). This comparison clearly demonstrates the necessity of alternative approaches for carbonyl detection from agar samples.

**Fig 1:**
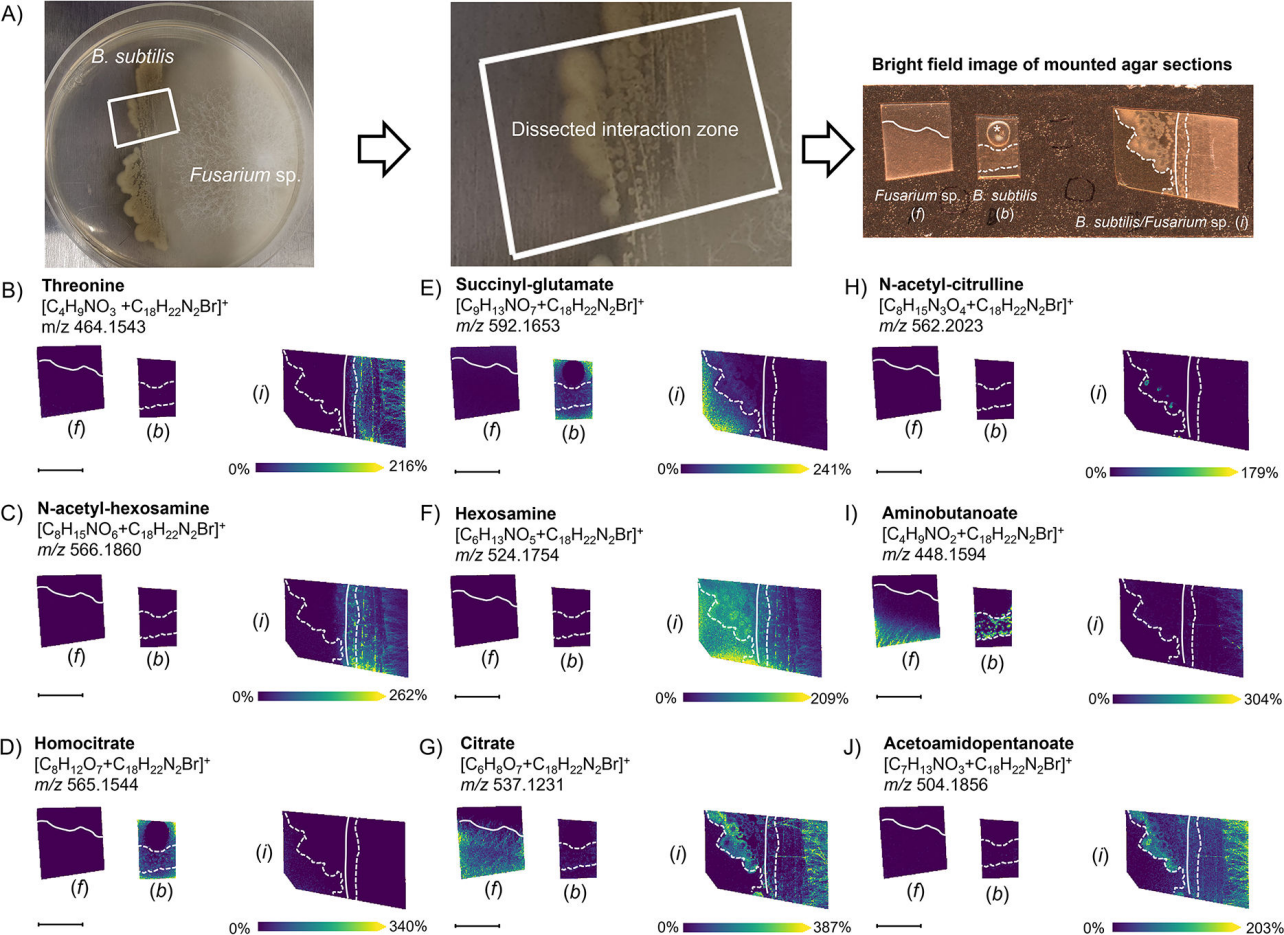
Characteristic patterns observed in the production and distribution of carbonyls from the interaction of *B. subtilis* and *Fusarium* sp. using the 4-APEBA-based OTCD approach. Each image is annotated with (*f*) showing the isolated *Fusarium* sp. control, (*b*) showing the isolated *B. subtilis* control, and (*i*) showing the interaction zone of the co-culture of *B. subtilis* and *Fusarium* sp. (**A**) Photograph of *B. subtilis* and *Fusarium* sp. in interacting on the agar plate, a zoomed view into the dissected interaction zone, and an optical image of isolated colonies and the interaction of *B. subtilis* and *Fusarium* sp. Colonies were mounted on the double-sided copper tape-covered slides. MALDI-MSI ion images of carbonyls are highlighted that show (B and C) increased production in *Fusarium* sp. while in interaction with *B. subtilis*, (**D**) suppressed excretion from *B. subtilis* while in interaction with *Fusarium* sp., (**E**) no change in abundance between isolated and colonies in interaction, (**F**) increased excretion from *B. subtilis* during interaction with *Fusarium* sp., (**G**) increased production in *B. subtilis* and steady state in *Fusarium* sp. during interaction, (**H**) hot spots in *B. subtilis* biofilm during interaction with *Fusarium* sp., (**I**) suppressed production in both *B. subtilis* and *Fusarium* sp. in interaction compared to isolated cultures, and (J) increased production in both *B. subtilis* and *Fusarium* sp. in interaction compared to isolated culture. Solid and dashed white lines on the ion images indicate the boundaries of *Fusarium* sp. and *B. subtilis* colonies, respectively. Scale bars are 7 mm, and each ion image intensity is respectively scaled. SMART annotation (30): S (step size, spot size, and total scans) = 100 µm, 30 µm × 30 µm, and 37,672 scans; M (molecular confidence) = MS1, 3 ppm; A (annotations) = 316 (METASPACE, KEGG [20% false discovery rate {FDR}], [M+C_18_H_22_N_2_Br]^+^); R (resolving power) = 110,000 at *m*/*z* 400; and T (time of acquisition) = 745 min.

In the 4-APEBA-based OTCD analysis, several spatial patterns that depict changes in specific metabolite production between isolated and interacting microbes were observed (Fig. 1). For example, we identified a group of molecules produced and excreted by *B. subtilis* only in its interaction with *Fusarium* sp. (e.g., hexosamine; Fig. 1F). Conversely, the production of an *N*-acetylated form of hexosamine (likely *GlcNAc*) was triggered in *Fusarium* sp. but not *B. subtilis*, with the interaction of these two species (Fig. 1C). This finding is congruent with previous reports that *GlcNAc* acts as a signal inducer within fungi and also serves in interkingdom communication (27). In this case, it is possible that fungi utilize hexosamine produced by *B. subtilis* for GlcNAc biosynthesis, but it is more likely that GlcNAc is released from the fungal cell wall (chitin) during the interaction (28). Contrasting spatial distributions were observed with citrate and homocitrate (Fig. 1G and D, respectively), which are two chemically and metabolically related molecules. We observed homocitrate to be produced only in isolated *B. subtilis* colonies, while citrate, a central metabolite of the tricarboxylic acid cycle (TCA) (29), was produced in both isolated and interacting *Fusarium* spp. Based on citrate co-localization with *B. subtilis* cells only in the interaction zone, we hypothesize that citrate originating from *Fusarium* sp. might serve as a carbon source for adjacent *B. subtilis* colonies during the interaction. Our MSI data also found that other carbonyls have a variety of unique spatial patterns, including those unchanged during interaction (e.g., succinyl-glutamate; Fig. 1E), present in distinct phenotypes of the *B. subtilis* biofilm (e.g., acetyl-citrulline; Fig. 1H), suppressed in both species when interacting (e.g., aminobutanoate; Fig. 1I), and activated in both species in their interactions (e.g., acetamidopentanoate; Fig. 1J). A list of all other metabolites annotated, together with their discriminant coefficients (areas under the curve [AUCs]) between isolated and interacting species, can be found in Table S1.

**Fig 2:**
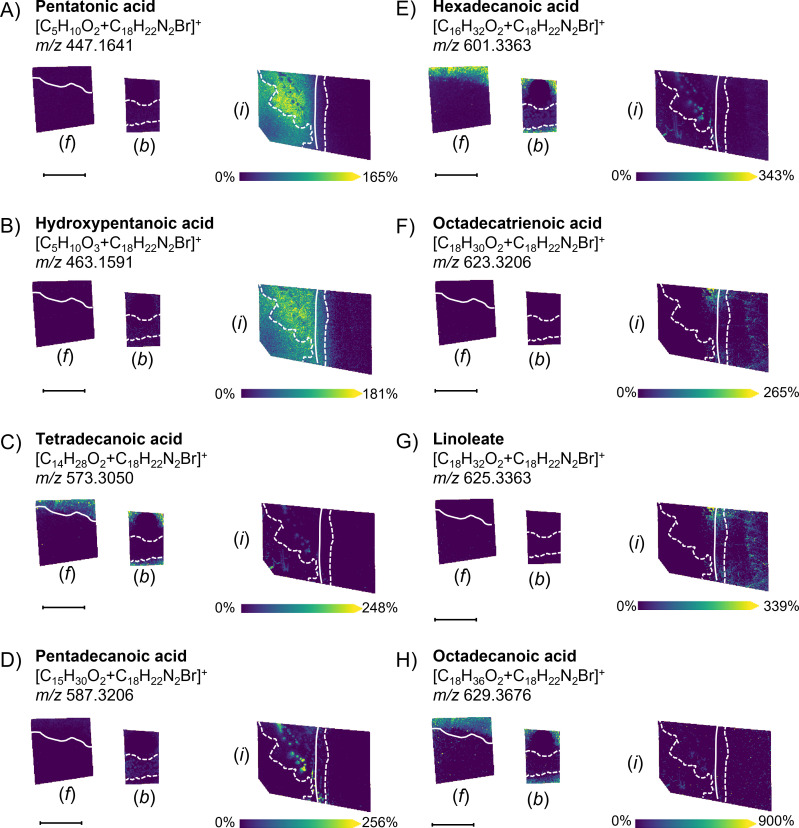
Shift in the production and distribution of select aliphatic carboxylic acids during the *B. subtilis* and *Fusarium* sp. interaction, as well as isolated controls of each culture using the 4-APEBA-based OTCD approach. (A) Pentatonic acid ion image, (B) hydroxypentatonic acid ion image, (C) tetradecanoic acid ion image, (D) pentadecanoic acid ion image, (E) hexadecanoic acid ion image, (F) octadecatrienoic acid ion image, (G) linoleate ion image, and (H) octadecanoic acid ion image. Each image is annotated with (*f*) showing the isolated *Fusarium* sp. control, (*b*) showing the isolated *B. subtilis* control, and (*i*) showing the interaction zone of the co-culture of *B. subtilis* and *Fusarium* sp. Solid and dashed white lines on the ion images indicate the boundaries of *Fusarium* sp. and *B. subtilis* colonies, respectively. Scale bars are 7 mm, and each ion image intensity is respectively scaled. SMART annotation (30): S (step size, spot size, and total scans) = 100 µm, 30 µm × 30 µm, and 37,672 scans; M (molecular confidence) = MS1, 3 ppm; A (annotations) = 316 (METASPACE, KEGG [20% FDR], [M+C_18_H_22_N_2_Br]^+^); R (resolving power) = 110,000 at *m*/*z* 400; and T (time of acquisition) = 745 min.

Besides homocitrate (Fig. 1D), citrate (Fig. 1G), and several dozen other polycarboxylic acids (Table S1), we annotated numerous aliphatic monocarboxylic acids (i.e., free fatty acids [FFAs]) as 4-APEBA derivatives (Fig. 2). Their spatial profile points to the different roles of these molecules during the *B. subtilis* and *Fusarium* sp. interaction. For example, there is intense excretion of short-chain FFAs (Fig. 2A and B) from *B. subtilis* during interaction with *Fusarium* sp. In contrast, medium-chain FFAs (C14–C16; Fig. 2C through E) are co-localized with *B. subtilis* cells further from the interaction zone. Strikingly, those with an even number of carbons (C14 and C16) are also highly excreted in the surrounding agar in isolated bacterial and fungal cultures, whereas C15 is not observed in any of the isolated cultures. It is known that *Bacillus* spp. can modify their FFA patterns to adapt to a wide range of environmental changes (31), and based on our results, it seems that the production of short-chain FFAs is vital for survival in a *Fusarium* sp. environment. On the other hand, *Fusarium* sp., in interaction with *B. subtilis*, boosts the production of unsaturated long-chain FFAs (C18, octadecatrienoic acid, and linoleate; Fig. 2F and G), while saturated long-chain FFAs seem to be more significantly excreted from isolated colonies than within interactions (Fig. 2H). This reinforced production of unsaturated long-chain FFAs might be related to the antimicrobial defense mechanisms of *Fusarium* sp. Namely, the antibacterial properties of FFAs are used by many organisms, where the prime target of FFA action is the bacterial cell membrane, where FFAs disrupt the electron transport chain and oxidative phosphorylation (32). Optical microscopy images of co-cultured *B. subtilis* NCIB 3610 and *Fusarium* sp. DS 682 demonstrate an antagonistic interaction, as there is reduced fungal growth in the presence of *B. subtilis* compared to the control (Fig. S2). *B. subtilis* is known to protect host plants by decreasing pathogenic fungal or bacterial growth through the production of secondary metabolites (33), and as the majority of these compounds contain carbonyls, 4-APEBA-based OTCD enables the sensitive tracing of their redistribution and kinetics.

**Fig 3:**
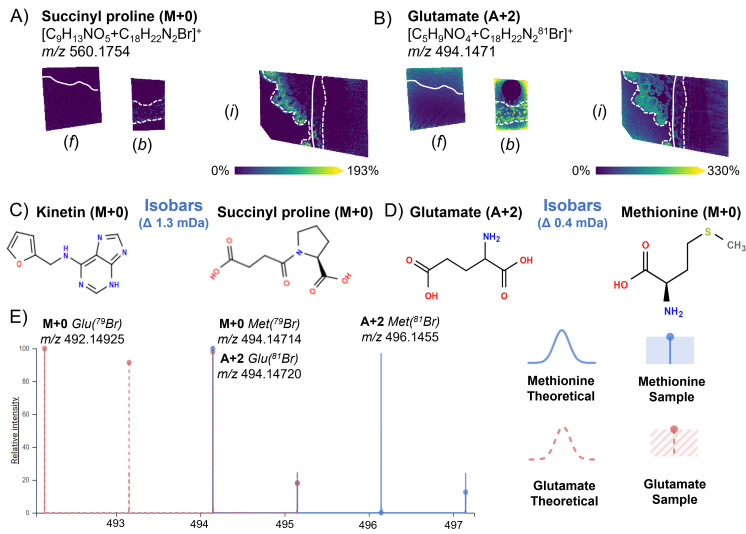
Resolving isobars using the 4-APEBA-based OTCD approach. Each image is annotated with (*f*) showing the isolated *Fusarium* sp. control, (*b*) showing the isolated *B. subtilis* control, and (*i*) showing the interaction zone of the co-culture of *B. subtilis* and *Fusarium* sp. (**A**) MALDI-MSI ion images of *m/z* 560.1754 are shown with (C) chemical structures of tentatively annotated derivatized isobaric metabolites monoisotopic kinetin (M+0, C_10_H_9_N_5_O+C_18_H_22_N_2_^79^Br) and monoisotopic succinyl proline (M+0, C_9_H_13_NO_5_+C_18_H_22_N_2_^79^Br), which differ by 1.3 mDa. Since kinetin does not contain a carbonyl that can be derivatized, this confirms the annotation of succinyl proline. (**B**) MALDI-MSI ion images of *m/z* 490.1471 are shown with (D) chemical structures of the tentatively annotated derivatized isobaric metabolite monoisotopic methionine (M+0, C_5_H_11_NO_2_S+C_18_H_22_N_2_^79^Br) and the second isotopologue glutamate (A+2, C_5_H_9_NO_4_+C_18_N_2_H_22_^81^Br), which differ by 0.4 mDa. (**E**) Theoretical simulations and the sample spectrum of isotopic distributions of both tentatively annotated isobars of methionine and glutamate from METASPACE, where within the sample spectrum, the monoisotopic methionine (M+0, ^79^Br) and the third (A+2, ^81^Br) isotopologue of methionine are not present at ratios representative of ^79^Br and ^81^Br isotopic distributions (blue trace vs blue dot). This confirms methionine as a false annotation of the third isotopologue (A+2, ^81^Br) of glutamate. Solid and dashed white lines on the ion images indicate the boundaries of *Fusarium* sp. and *B. subtilis* colonies, respectively. Scale bars are 7 mm, and each ion image intensity is respectively scaled. SMART annotation (30): S (step size, spot size, and total scans) = 100 µm, 30 µm × 30 µm, and 37,672 scans; M (molecular confidence) = MS1, 3 ppm; A (annotations) = 316 (METASPACE, KEGG [20% FDR], [M+C_18_H_22_N_2_Br]^+^); R (resolving power) = 110,000 at *m*/*z* 400; and T (time of acquisition) = 745 min.

Besides comprehensive carbonyl coverage, the additional value of this approach is the ability to confidently resolve some isomeric and isobaric metabolites (Fig. 3) that are undistinguishable in typical MALDI-MSI experiments, which only provides accurate mass measurements without gas phase separation by ion mobility. Figure 3A shows a MALDI-MSI ion image of a brominated derivative product ion at *m*/*z* 560.1754, illustrating that this metabolite is concentrated on the *B. subtilis* biofilm layer further from the interaction zone. Based on the accurate mass measurements, this ion, within a 3-ppm window, can be ascribed to the derivatized forms of either kinetin (C_10_H_9_N_5_) or succinyl proline (C_9_H_13_NO_5_) (Fig. 3C). Both molecules are naturally present in soil (e.g., as a plant hormone and common amino acid, respectively). Since kinetin does not possess a carbonyl group that can be derivatized with 4-APEBA, it suggests that this ion corresponds to succinyl proline, as succinyl proline contains multiple carbonyl groups (Fig. 3C). Interestingly, upregulation of succinylation is a known phenomenon in the *B. subtilis* response to carbon source changes, especially when citrate becomes the carbon source (34). This succinylation-citrate relationship orthogonally validates our previous hypothesis that *Fusarium* sp. produces citrate (Fig. 1G), which is further metabolized by *B. subtilis*.

The second example of resolving isobars with 4-APEBA-based OTCD illustrates the importance of using bromine as a non-leaving moiety of the derivatization agent. Namely, bromine has two stable isotopes (^79^Br and ^81^Br) with similar relative abundances (51% and 49%, respectively), producing an easily recognizable isotopic pattern, where the monoisotopic (M+0; ^79^Br) and second isotopologue (A+2; ^81^Br) peaks have similar intensities. For instance, the ion at *m*/*z* 494.1471 (Fig. 3B) can be wrongly annotated as methionine (M+0; ^79^Br derivative) but is actually an isotopologue of glutamate (A+2; ^81^Br derivative). If this were methionine (Fig. 3D), an A+2 isotopologue at *m*/*z* 496.1455 would have a similar intensity as the putative monoisotopic peak at *m*/*z* 494.1471 (Fig. 3E), which was not the case. Instead, the peak at *m*/*z* 492.1492 has the same intensity and exact spatial localization as *m*/*z* 494.1471, indicating glutamate was derivatized.

**Fig 4:**
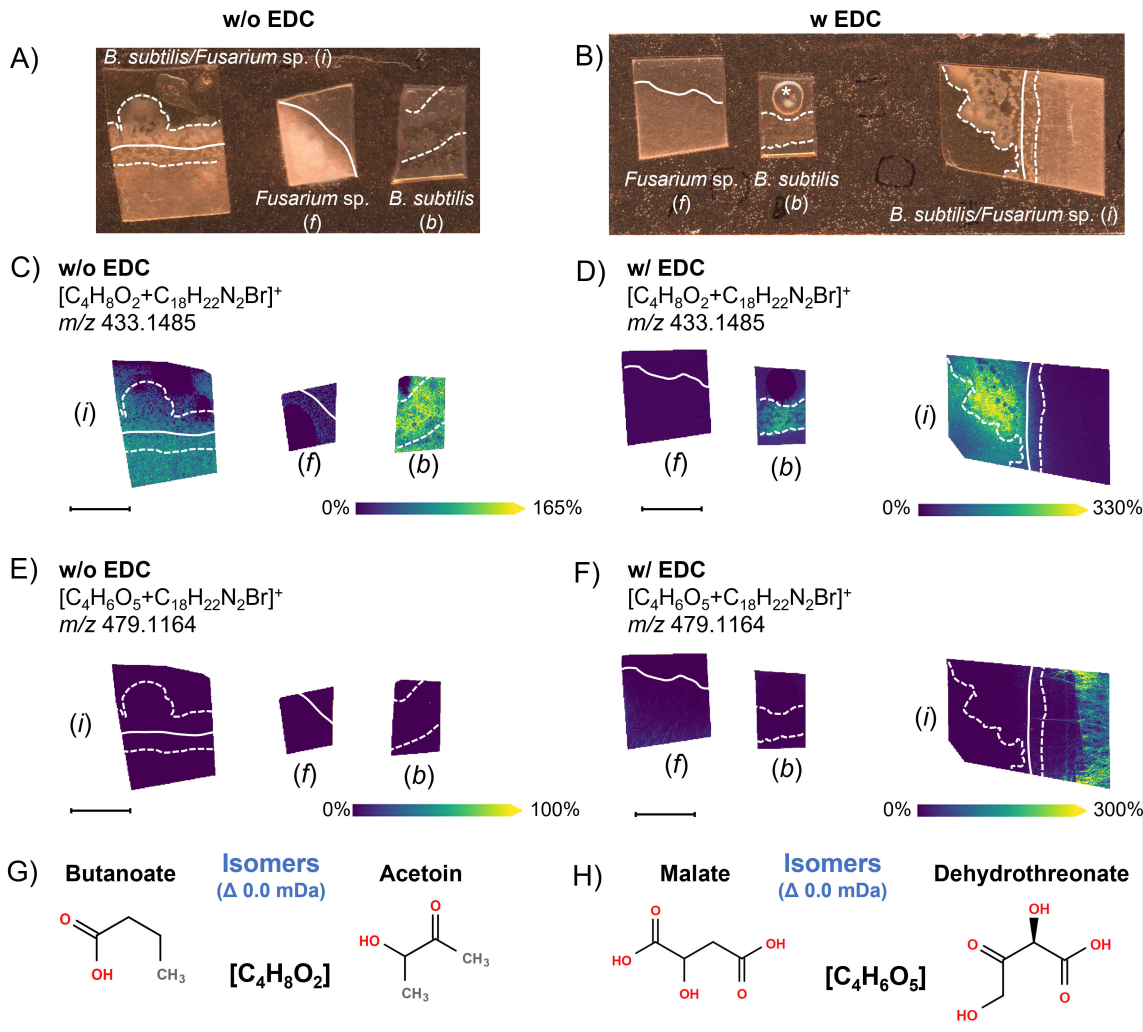
Resolving isomers using 4-APEBA-based OTCD with and without prior addition of EDC, which activates carboxylic acids prior to derivatization. Each image is annotated with (*f*) showing the isolated *Fusarium* sp. control, (*b*) showing the isolated *B. subtilis* control, and (*i*) showing the interaction zone of the co-culture of *B. subtilis* and *Fusarium* sp. Brightfield images from samples that underwent preparation (A) without EDC, as shown within Fig. 1A, and (B) with EDC. MALDI-MSI ion images are shown for *m*/*z* 433.1485 (C) without EDC and (D) with EDC. Additional MALDI-MSI ion images of *m*/*z* 479.1164 are shown (E) without EDC and (F) with EDC. Here, the ion at *m/z* 479.1164 was not detected within (E) without prior activation of carboxylic acids with EDC. (**G**) The molecular structures of two tentatively annotated isomers, butanoic acid (carboxylic acid) and acetoin (ketone), are possible annotations for *m*/*z* 433.1485. (**H**) The molecular structures of the other two tentatively annotated isomers, malate (solely carboxylic acid) and dehydrothreonate (oxoacid), are depicted for annotations at *m/z* 479.1164. Solid and dashed white lines on the ion images indicate the boundaries of *Fusarium* sp. and *B. subtilis* colonies, respectively. Scale bars are 7 mm, and each ion image intensity is respectively scaled. SMART annotation (30): S (step size, spot size, and total scans) = 100 µm, 30 µm × 30 µm, and 37,672 scans; M (molecular confidence) = MS1, 3 ppm; A (annotations) = 316 (METASPACE, KEGG [20% FDR], [M+C_18_H_22_N_2_Br]^+^); R (resolving power) = 110,000 at *m*/*z* 400; and T (time of acquisition) = 745 min.

Lastly, the two-step 4-APEBA-based OTCD approach could be utilized to also differentiate some carbonyl isomers (Fig. 4). This is because if 4-APEBA is used alone (35), then it can only derivatize aldehydes and ketones, and it will not derivatize carboxylic acids (21). EDC (1-ethyl-3-(3-dimethylaminopropyl)carbodiimide) must be used as an activator for 4-APEBA to derivatize carboxylic acids (Fig. S3) (36, 37). As such, we performed 4-APEBA-based OTCD with and without EDC on a replicate sample. Multiple overlapping and unique annotations were observed with side-by-side annotation outputs, and ion images are visualized in METASPACE (38) and Table S2. Overlapping annotations between both conditions (with and without the application of EDC) indicate the presence of a ketone or aldehyde group in the metabolite. Whereas in the case in which the annotation was only present when EDC was applied prior to 4-APEBA, annotated molecules contain solely carboxylic acids, and aldehyde or ketone groups are absent from their structure and should not be considered. One example of an ambiguous annotation is the ion image at *m/z* 433.1485 (C_4_H_8_O_2_) (Fig. 4C and D). This molecular formula can correspond to two natural products of bacterial metabolism: butanoic acid and acetoin (3-hydroxy-2-butanone) (Fig. 4G). As a similar spatial pattern was observed with the addition of EDC (Fig. 4C) and without the addition of EDC (Fig. 4D), this ion image, which indicates intense excretion of the metabolite from *B. subtilis*, is likely the ketone, acetoin, because the carboxylic acid, butanoic acid, cannot be derivatized without EDC. This annotation aligns well with the fact that acetoin is a primary catabolic product of *B. subtilis,* which bacteria reuse during the stationary phase when other carbon sources have been depleted (39). Moreover, the ion image at *m*/*z* 447.1641 with a similar spatial pattern was previously annotated as pentanoic acid (Fig. 2A) and was not detected in the analysis without EDC (36), which orthogonally confirms the absence of butanoic acid in the analyzed sample. Other aliphatic carboxylic acids discussed throughout the manuscript (i.e.*,* FFAs and some components of the TCA cycle) were also annotated only if EDC was used as an activator, while oxocarboxylic acids, due to the presence of ketone or aldehyde groups, were annotated without EDC as well (Table S2). For example, the ion at *m*/*z* 479.1176 (Fig. 4E and F) with the molecular formula of C_4_H_6_O_5_ is malate (two carboxyl groups, no ketone or aldehyde groups) rather than dehydrothreonate (carboxyl and ketone groups), as this ion was not detected without EDC treatment (Fig. 4H).

**Fig 5:**
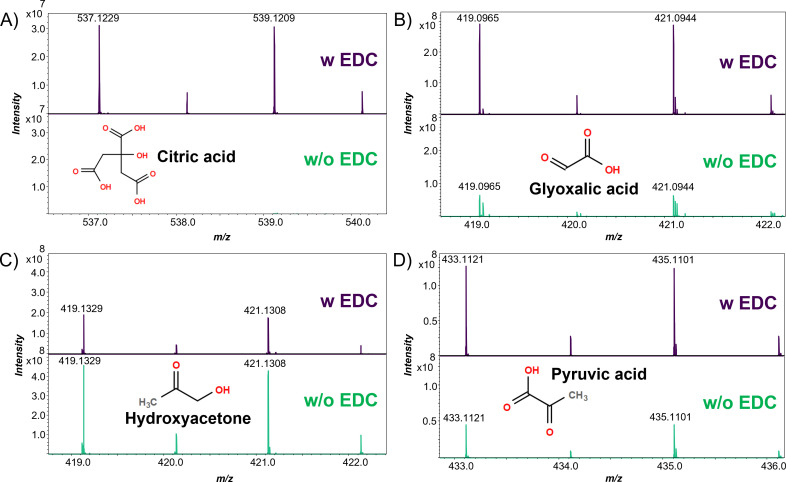
MALDI mass spectra of carbonyl standards with and without EDC activation prior to 4-APEBA derivatization. Mass spectra are shown for (A) citric acid as a model carboxylic acid, (**B**) glyoxalic acid as an oxocarboxylic acid with an aldehyde, (**C**) hydroxyacetone as an oxoacid with a ketone, and (D) pyruvic acid as an oxocarboxylic acid with a ketone. Peaks that correspond to the monoisotopic monoisotopic (M+0, ^79^Br) and the third isotopologue (A+2, ^81^Br) were annotated and shown in each spectrum, and the structure of each metabolite is also presented.

This EDC-guided selectivity of 4-APEBA was additionally confirmed by analysis of several standards with different chemistries. Specifically, we have citric acid, which solely has carboxyl groups; glyoxalic acid, which has a carboxylic and aldehyde group; hydroxyacetone, which has only a ketone group (analog to acetoin); and pyruvic acid, which has a carboxylic and ketone group (Fig. 5). Our results show that citric acid, with no ketone or aldehyde groups, was not derivatized without activation by EDC (37). Due to the presence of a ketone group in pyruvic acid and an aldehyde group in glyoxalic acid, these oxoacid metabolites were derivatized without EDC, although with ~50% lower signal intensities than with EDC. This difference in intensity could be a consequence of matrix heterogeneity in dried droplet preparations but might also be the result of different derivatization efficiencies between the two treatments. In fact, hydroxyacetone, the demethylated analog of acetoin, which we previously distinguished from its isobar butanoic acid (Fig. 4G), shows clear isotopic patterns corresponding to its 4-APEBA derivatized product, and its signal is twofold higher without EDC than with EDC. Therefore, we hypothesize that there are different kinetics and equilibria in the derivatization of ketoacids and ketones by 4-APEBA, which can be exploited further for their differentiation. Importantly, none of the analytical standards show evidence of double or multiple derivatizations in any MALDI-Fourier-transform ion cyclotron resonance (FTICR) mass spectra presented.

### Conclusions

Mapping the redistribution of acidic compounds in the microbial ecosystem using MALDI-MSI is a significant bioanalytical challenge. This challenge is further exacerbated for microbial cultures on agar because many of these metabolites readily ionize in negative ionization mode, which is not suitable for this type of sample. This work demonstrates a new approach for spatially profiling a diverse set of carbonyls, from small volatile aldehydes, ketones, and short-chain FFAs to long-chain aliphatic carboxylic acids, including lactones and oxocarboxylic acids, directly from microbial cultures using 4-APEBA-based OTCD and MALDI-MSI. From our proof-of-concept experiments, we were able to map and reveal the distribution of carbonyls resulting from the interaction of *B. subtilis* and *Fusarium* sp. cultured on agar. These results illustrate the potential role of citrate, hexosamines, and FFAs in this interaction. The additional advantage of the described OTCD by 4-APEBA is that it can distinguish some isobaric and isomeric species, especially those with only a carboxyl group and only a ketone or aldehyde group, as well as those that do not have said functional groups at all. This benefit provides greater confidence in the biological interpretation of microbial MSI data. We also envision the application of this workflow to map metabolic processes across a broad range of microbiology fields. For example, it can be used to spatially resolve the metabolome of digestive processes of the human gut, where microbiota produce large quantities of aliphatic acids and aldehydes through anaerobic fermentation of dietary fibers, which influences gut-brain communication and brain function (40). Another potential use is in resolving temporal-spatial chemistry of microbiological lignocellulose decay mechanisms where aldehydes, ketones, and carboxylic acids are both main degradation products and enzyme mediators for further lignin decomposition (41).

## MATERIALS AND METHODS

### Microbial growth and co-culture preparation

*B. subtilis* NCIB 3610 was cultured as described before (42). Briefly, the strain was cultured on MSgg, a *B. subtilis* biofilm-promoting medium: 5  mM potassium phosphate (pH 7) (Fisher Scientific, Waltham, MA, USA), 100  mM MOPS (morpholinepropanesulfonic acid [pH 7]; Sigma-Aldrich, St. Louis, MO, USA), 2  mM MgCl_2_ (Fisher Scientific, Waltham, MA, USA), 700  µM CaCl_2_ (Alfa Aesar, Haverhill, MA, USA), 50  µM MnCl_2_ (Fisher Scientific, Waltham, MA, USA), 50  µM FeCl_3_ (Sigma-Aldrich, St. Louis, MO, USA), 1  µM ZnCl_2_ (Sigma-Aldrich, St. Louis, MO, USA), 2  µM thiamine hydrochloride (Fisher Scientific, Waltham, MA, USA), 0.5% glycerol (Fisher Scientific, Waltham, MA, USA), and 0.5% glutamate (Sigma-Aldrich, St. Louis, MO). Next, 1.5% agar (BD) was added to the medium to prepare agar plates using MSgg media, and approximately 5 mL of MSgg agar per plate was poured. MSgg agar plates were streaked with *B. subtilis* from frozen stocks and incubated at 28°C for 15 h.

*Fusarium* sp. DS 682 was cultured as described before (43). Fungi were maintained on potato dextrose agar (PDA) plates at 28°C. For co-culture experiments, a disposable 1.5-mm hole punch (Integra Miltex, York, PA, USA) was used to collect the fungal biomass from a fungal culture PDA agar plate (one agar plug with fungi) and was placed on the MSgg agar plate. Fungi were grown at 28°C for 5 days. After 5 days of fungal growth, *B. subtilis* was streaked close to the fungal biomass as described above. Agar plates were prepared for MSI analysis after 2 days of bacterial growth at 28°C.

Agar areas with isolated and interacting colonies were excised from MSgg agar Petri dishes, placed onto double-sided adhesive copper tape (3-6-1182; 3M United States) that adhered to indium tin oxide-coated glass slides (Bruker Daltonics, Billerica, MA, USA), and dried at room temperature (RT) overnight prior to derivatization and analysis (Fig. 1A).

### *In situ* chemical derivatization and MALDI matrix application

Agar samples were chemically derivatized by either sole application of synthesized 4-APEBA at 2 mg/mL or with a two-step approach: spraying an aqueous solution of EDC (Sigma-Aldrich, St. Louis, MO, USA) at 6 mg/mL first with subsequent application of 4-APEBA at 2 mg/mL using an external syringe pump with the M5-Sprayer (HTX Technologies, Chapel Hill, NC, USA). Spraying parameters were the same for both chemicals: a 25 µL/min flow rate, a nozzle temperature of 37.5°C, four cycles at 3-mm track spacing with a crisscross pattern, a 2-s drying period, 1,200 mm/min spray head velocity, 10 PSI of nitrogen gas, and a 40-mm nozzle height. DHB (2,5-dihydroxybenzoic acid; Sigma-Aldrich, St. Louis, MO, USA) was prepared at a concentration of 40 mg/mL in 70% MeOH and was sprayed at a 50 µL/min flow rate using the same M5-Sprayer. The nozzle temperature was set to 70°C, with 12 cycles at 3-mm track spacing with a crisscross pattern. A 2-s drying period was added between cycles, and a linear flow was set to 1,200 mm/min with 10 PSI of nitrogen gas and a 40-mm nozzle height. This resulted in a matrix coverage of ~667 µg/cm^2^ for DHB. For negative ion mode experiments, we used NEDC (Sigma-Aldrich, St. Louis, MO, USA), which in our and other laboratories (44) yields more endogenous compounds than other commonly used matrices for negative mode analyses, such as 9-AA or 1,5-DAN. NEDC was prepared at a concentration of 7 mg/mL in 70% MeOH and was sprayed at a 120 µL/min flow rate using the same M5-Sprayer. The nozzle temperature was set to 70°C, with eight cycles at 3-mm track spacing with a crisscross pattern. A 0-s drying period was added between cycles, and a linear flow was set to 1,200 mm/min with 10 PSI of nitrogen gas and a 40-mm nozzle height. This resulted in a matrix coverage of ~187 µg/cm^2^ for NEDC.

### Derivatization of analysis of standards with and without EDC addition

Standards of citric acid (Sigma-Aldrich, St. Louis, MO, USA), glyoxalic acid (Sigma-Aldrich, St. Louis, MO, USA), hydroxyacetone (Sigma-Aldric, St. Louis, MO, USA), and pyruvic acid (Sigma-Aldrich, St. Louis, MO, USA) were prepared by dissolving each of them individually in Milli-Q water to a final concentration of 0.01 mg/mL. For each EDC/4-APEBA reaction, 10 µL of each standard was diluted in 400 µL of 6 mg/mL EDC and 400 µL of 2 mg/mL 4-APEBA. For each 4-APEBA reaction without EDC, each standard was diluted in 400 µL of Milli-Q water and 400 µL of 2 mg/mL 4-APEBA. Reactions were quenched after 2 h, and 1 µL of each reaction was spotted onto a MALDI MTP 384 target plate (Bruker Daltonics, Billerica, MA, USA) and mixed with 1 µL of DHB matrix (40 mg/mL in 70% MeOH).

### MALDI-MSI analysis and data processing

All imaging and analyses of standards were performed on a Bruker Daltonics 12T solariX FTICR MS equipped with a ParaCell, Apollo II dual ESI, and MALDI source with a SmartBeam II frequency-tripled (355 nm) Nd:YAG laser (Bremen, Germany). Positive ion mode OTCD and negative ion mode NEDC acquisitions were acquired with broadband excitation from *m*/*z* 98.3 to 1,000, resulting in a detected transient of 0.5593 s—the observed mass resolution was ~110k at *m*/*z* 400. FlexImaging (Bruker Daltonics, v.5.0) was used for the imaging experiments, and analyses were performed with a 100-µm step size. FlexImaging sequences were directly imported into SCiLS Lab (Bruker Daltonics, v.2023.a Premium 3D) using automatic magnetic resonance mass spectrometry (MRMS) settings. Ion images were directly processed from the profile data sets within SCiLS Lab, and automated annotation of the centroided data set was completed within METASPACE with a chemical modifier corresponding to the mass shift expected from 4-APEBA derivatization (+C_18_H_22_N_2_Br, +345.0966 Da). KEGG-v1 was used as a metabolite database for annotations. All annotations were imported back to SCiLS as a new peak list, and discrimination analysis (receiver operating characteristic ROC]) between species in the interaction and corresponding isolated species was performed on that list. The AUC of ROC analysis for each pair (isolated microbe vs microbe in interaction) was calculated.

## Data Availability

All MALDI-MSI data sets and annotations are publicly available at METASPACE: https://metaspace2020.eu/project/velickovic-2023

## SUPPLEMENTAL MATERIAL

Supplemental figures msystems.00803-23-s0001.docx Fig. S1 to S3.

Table S1 msystems.00803-23-s0002.xlsx Discriminate analyses completed for co-cultured *B. subtilis* and *Fusarium* sp. highlighting AUCs within the isolated colonies and the interaction zone.

Table S2 msystems.00803-23-s0003.xlsx All annotations from KEGG-v1 output of METASPACE for experiments with and without application of EDC before 4-APEBA.

ASM does not own the copyrights to Supplemental Material that may be linked to, or accessed through, an article. The authors have granted ASM a non-exclusive, world-wide license to publish the Supplemental Material files. Please contact the corresponding author directly for reuse.
